# Metastatic multicentric epithelioid angiosarcoma of bone. A case report with pitfalls. Tumor seeding after percutaneous biopsy and hypercalcemia

**DOI:** 10.1093/jscr/rjae022

**Published:** 2025-01-07

**Authors:** Jorge Fuentes-Sánchez, Eva Manuela Pena-Burgos, Mar Tapia-Viñe, Jose Juan Pozo-Kreilinger, Eduardo Jose Ortiz-Cruz

**Affiliations:** Orthopedic Oncologist Unit, La Paz University Hospital, P° Castellana 261, 28046 Madrid, Spain; Pathology Department, Príncipe de Asturias University Hospital, Av. Principal de la Universidad, 1, 28805 Alcalá de Henares, Madrid, Spain; Musculoskeletal Radiology Unit Department, La Paz University Hospital, P° Castellana 261, 28046 Madrid, Spain; Pathology Department, La Paz University Hospital, P° Castellana 261, 28046 Madrid, Spain; Orthopaedic Oncologist Unit, La Paz University Hospital, P° Castellana 261, 28046 Madrid, Spain

**Keywords:** angiosarcoma, multicentric, metastasis, core needle biopsy, hypercalcemia, tumor seeding

## Abstract

Angiosarcomas are a type of malignant tumor of vascular origin. They represent <1% of all primary bone tumors. The multicentric or metastatic does not differ in its high aggressiveness and poor prognosis. A 72-year-old male with bone angiosarcoma initially located in proximal femur. After biopsy in a non-expert sarcoma center, he presented tumor involvement in the needle trajectory and multicentric/metastatic involvement at the sacro-coccygeal level. He associated tumoral-hypercalcemia and was referred to our sarcoma center. He was treated by tumoral resection and tumor prosthesis. In the follow-up he presented pulmonary metastasis and new implants, dying 2 months later. Multicentric or metastatic bone involvement in angiosarcomas has only theoretical relevance for their management. Biopsy should be performed in sarcoma centers due to the risk of dissemination. Although hypercalcemia in sarcomas is uncommon, we highlight its investigation.

## Introduction

Angiosarcomas are a high-grade malignant neoplasm with endothelial differentiation [1]. Although they can arise in virtually any organ of the body, bone tissue involvement is extremely rare, ~3.6% of all angiosarcomas [2].

It appears more frequently in adults [3, 4]. In most cases they are single tumors, but sometimes they present as a multifocal disease, which can be difficult to distinguish from bone metastases [3, 5, 6]. They are particularly aggressive with a high rate of lymph node involvement and local recurrence [7]. The delay in diagnosis is usually due to its insidious growth, in which biopsy becomes the last step of certainty for histological and immunohistochemical analysis [7–9]. Core needle biopsy (CNB) is the most used technique in sarcoma centers [10] and its treatment is mainly surgical [3, 4].

In this illustrative case of multicentric bone angiosarcoma with subsequent pulmonary metastasis we analyze the possible reasons of its dissemination during the diagnostic process after percutaneous biopsy. In addition, a review of this rare tumor is made.

## Case-report

A 72-year-old male debuted 3 months earlier with pain in the proximal region of the left thigh without associated trauma.

**Figure 1:**
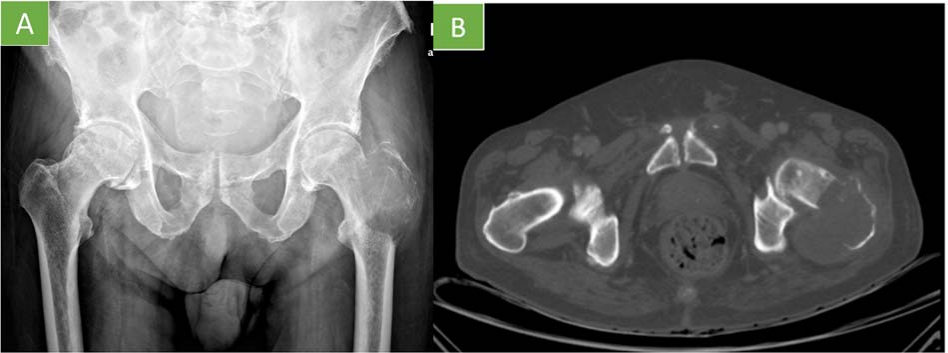
(A) X-ray pelvis AP view: geographic lytic insufflating expansive lesion centered in the intertrochanteric region of the left femur that destroys the cortex with poorly defined borders in certain locations. Soft tissue mass associated. The tumor matrix shows thin septum reaching 9 cm in longitudinal axis. (B) Axial CT: lytic lesion with cortical destruction at trochanteric level.

The radiological image showed an osteolytic lesion that had destroyed the intertrochanteric region of the left femur (Fig. 1A). A computed tomography (CT) was performed and showed the femur lesion with an associated soft tissue mass (Fig. 1B). In the staging studies, other detectable metastases were ruled out.

Due to the intense pain, he was admitted to hospital. During admission he presented hypercalcemia (maximum corrected calcium level, 14.5 mg/dL). After evaluation by the internal medicine service, malignant hypercalcemia secondary to osteolytic lesion and humeral mechanism (HHM) was evidenced. In order to obtain a sample of the bone lesion, a percutaneous CNB was performed in the region posterior to the greater trochanter with a pathological diagnosis of angiosarcoma. He was subsequently referred to our sarcoma center for a therapeutic decision.

Considering the high risk of pathological fracture with 12 points on the Mirels scale [8], the surgical treatment was indicated after evaluation by the Bone and Soft Tissue Tumors Committee (BSTTC).

**Figure 2:**
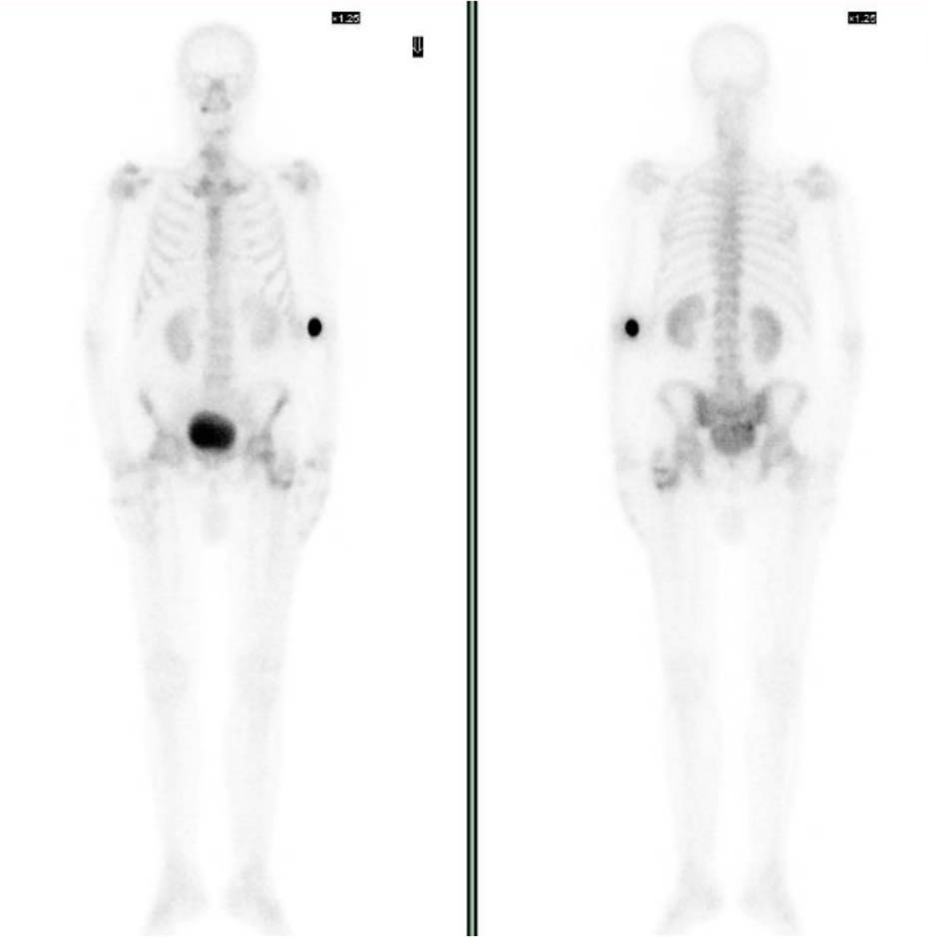
Bone scintigraphy of the entire skeleton with Tc99 and SPECT–CT. Highlighting of radioactive tracer in left proximal femur and sacrococcygeal region.

**Figure 3:**
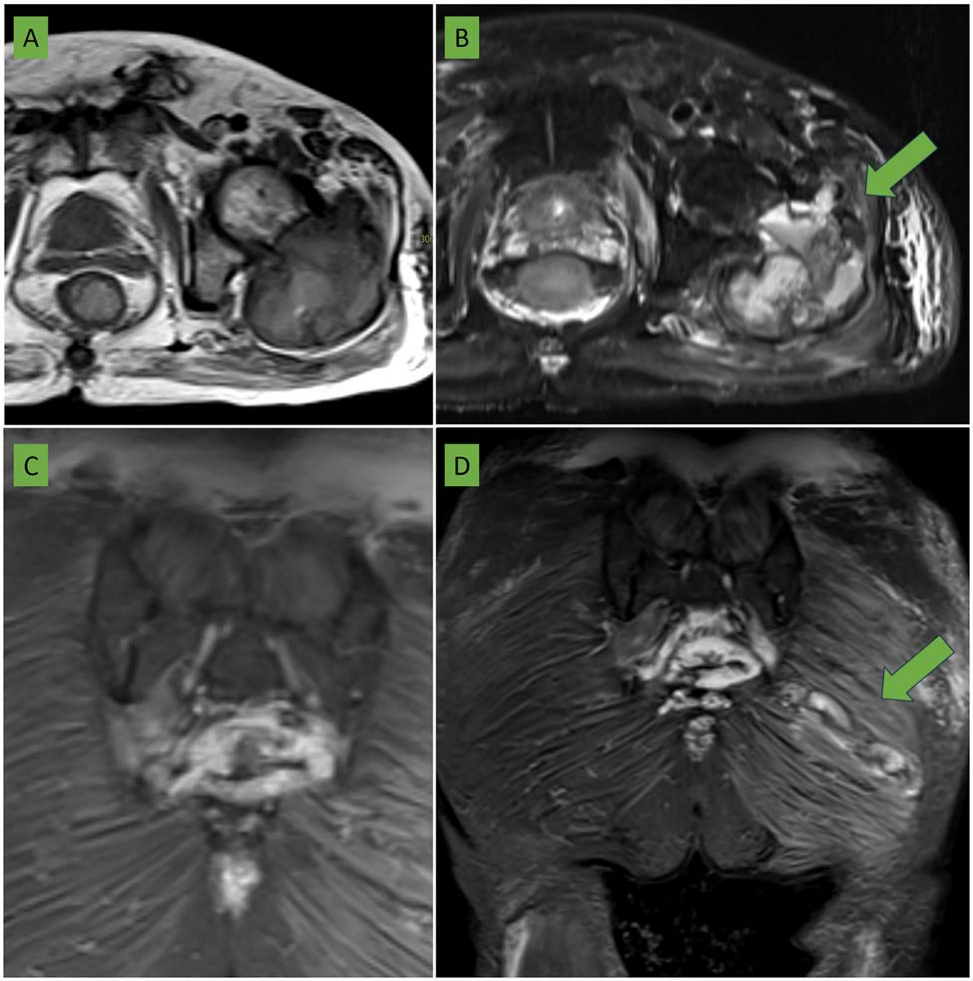
(A) T2 sequence with fat saturation and (B) T1-weighted sequence in axial plane centered on the left hip: a large expansive lesion is visualized centered in the trochanteric region extending to the femoral neck with a heterogeneous matrix of hyperintense predominance with respect to the muscle in both sequences in which cavities with liquid/liquid levels are identified (arrow) compatible with bleeding in different phases. The anterior cortex is focally rotated extending to the medial gluteal musculature (arrow). (C) Coronal section in STIR sequence showing sacrococcygeal involvement of the same characteristics as the femoral lesion. (D) Coronal section in STIR sequence centered in the gluteal region: an elongated lesion with lobulated borders is seen extending from the lateral region of the gluteus maximus to the proximity of the sacrococcygeal region (arrow). It coincides with the biopsy trajectory and is suggestive of tumor seeding.

Bone scintigraphy of the entire skeleton, SPECT–CT of the pelvis (Fig. 2) and magnetic resonance imaging (MRI) was performed for surgical planning (Fig. 3A–C).

In addition to previous lesion, an intramuscular soft tissue mass located in the left gluteus maximus not detected in the CT scan prior to the percutaneous biopsy, shows a probable tumor seeding in the trajectory towards the sacral region (Fig. 3D).

**Figure 4:**
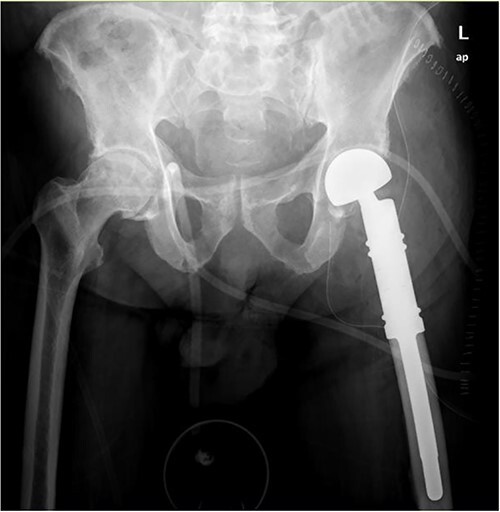
Wide intra-articular tumor resection of the proximal femur and reconstruction with tumor prosthesis and bipolar head.

Wide intra-articular tumor resection of the proximal femur and reconstruction with tumor prosthesis and bipolar head were indicated (Fig. 4).

**Figure 5:**
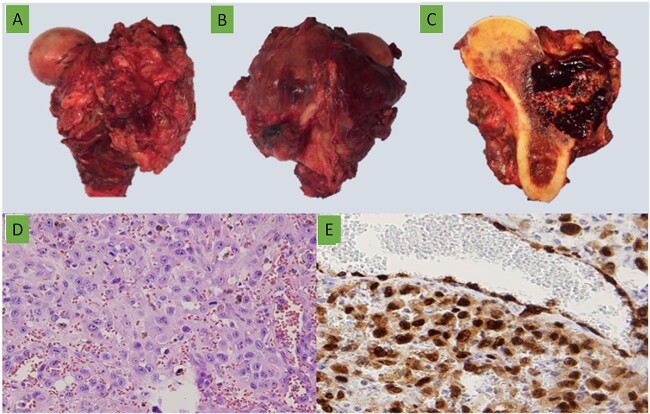
15 cm long of (A) anterior and (B) posterior gross views of the proximal femoral resection with a lateral soft tissue mass. (C) Lateral metaphysis-diaphyseal tumor with soft tissue extension, composed by bloody multiloculated cavities. (D) Epithelioid cells forming vascular channels with marked atypia (H&E ×400). (E) ERG (ERG ×400).

No macroscopic tumoral invasion of surgical margin was observed (Fig. 5A–C). The diagnosis of epithelioid angiosarcoma of bone was confirmed (Fig. 5D and E).

**Figure 6:**
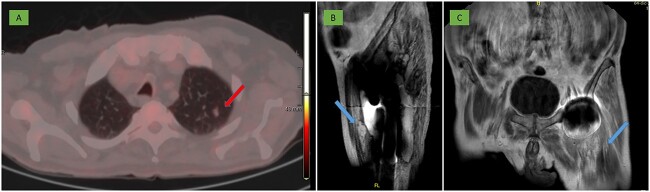
(A) PET-CT: 10 mm nodule in the left pulmonary apex with incipient metabolic activity suggestive of malignancy (arrow). (B, C) MRI: deep residual lesional focus in the anterolateral region dependent on the anterior rectus muscle (arrow).

After surgery, a nodule compatible with pulmonary metastasis was identified on PET-CT (Fig. 6A) and on MRI other similar metastases were also observed adjacent to the gluteus maximus and in the anterior rectus muscle (Fig. 6B and C).

Adjuvant therapies were not indicated by the BSTTC due to progressive clinical deterioration. A palliative care was assessed at the request of the patient and family, and he died 2 months after surgery.

## Discussion

Angiosarcomas are extremely rare primary bone tumors that accounts <1% of all malignant bone tumors [1]. It mainly affects the long bones, femur and tibia being the most frequently affected, followed by the axial skeleton. It presents two peaks of incidence in the second and seventh decade of life [3, 5].

Tumor-induced hypercalcemia is a relatively common complication of certain types of cancer, but has rarely been described in sarcoma patients [11]. The most common mechanism is HHM resulting from overproduction of PTHrP. It is estimated that ~80% is mediated by HHM, while almost 20% is the result of local osteolytic hypercalcemia. In our case, both mechanisms are involved. Regardless of the mechanism hypercalcemia in sarcomas is a rare disorder, which highlights the peculiarity of this tumor [12, 13].

Therapeutic options in angiosarcomas are scarce and are mainly based on surgical treatment [4, 5]. Life expectancy can be increased with early diagnosis and optimal surgery, which is not always possible [3].

CNB is the most commonly used technique for a histological and immunohistochemical study essential for diagnosis [7–10]. In the case presented, the initial biopsy performed generated a plausible tumor dissemination. Traditionally, in musculoskeletal oncology, it was assumed that the biopsy tract should be resected along with the tumor. Moreover, it has been described that recurrence-free survival is greater in patients without tumor seeding despite resection. According to Barrientos et al 107 months free of local recurrence compared to 11 months [14].

CNB should be performed by radiologists specialized in musculoskeletal pathology using a coaxial technique [15]. The location of the biopsy area has to be discussed with the orthopedic oncology surgeon according to the surgical approach. In the case of the proximal femur, it is generally performed over the greater trochanter, avoiding the gluteal muscles. Therefore, the lateral approach would probably have simplified access and reduce the risk of muscle contamination, favoring subsequent surgery for resection of the proximal femur [15].

In case descriptions, it is very difficult to know whether a patient with multiple lesions are metastases or a multifocal tumor, and this leads us to question the relevance of the multifocal bone angiosarcoma entity described in the literature and to suggest instead that the other lesions were metastases from a primary angiosarcoma. These findings highlight the rationale for whole-body diagnostic staging in this type of tumor, but we do not believe that they change the poor clinical course of the disease. Thariat *et al*. [6] indicate that in the presence of an angiosarcoma diagnosed as multifocal, the bone lesions may be metastases from an occult primary angiosarcoma, justifying a whole-body diagnostic workup. This analysis is consistent with that provided by Palmerini *et al*. [5], who evaluate multifocal bone lesions and metastases interchangeably. Therefore, this discussion has only theoretical relevance in our case.

The aim of the surgery was to improve the quality of life and avoid a pathological fracture, discouraging the resection at the sacrococcygeal level and therefore of the biopsy tract since his prognosis was not going to change. The final finding of pulmonary metastasis is consistent with the literature where 32% have metastases of which 19% are pulmonary [3].

Multicentric or metastatic epithelioid bone angiosarcomas are very rare tumors with a high aggressiveness and poor prognosis. They require diagnostic and therapeutic thoroughness to improve the patient’s quality of life and survival.

CNB have a risk of tumor seeding, so their approach must be evaluated to achieve maximum success in the diagnostic-therapeutic process.
